# Myasthenia Gravis: Autoantibody Specificities and Their Role in MG Management

**DOI:** 10.3389/fneur.2020.596981

**Published:** 2020-11-30

**Authors:** Konstantinos Lazaridis, Socrates J. Tzartos

**Affiliations:** ^1^Department of Immunology, Hellenic Pasteur Institute, Athens, Greece; ^2^Tzartos NeuroDiagnostics, Athens, Greece; ^3^Department of Neurobiology, Hellenic Pasteur Institute, Athens, Greece

**Keywords:** autoimmunity, myasthenia gravis, autoantibody, diagnosis, therapy, acetylcholine receptor, MuSK, LRP4

## Abstract

Myasthenia gravis (MG) is the most common autoimmune disorder affecting the neuromuscular junction, characterized by skeletal muscle weakness and fatigability. It is caused by autoantibodies targeting proteins of the neuromuscular junction; ~85% of MG patients have autoantibodies against the muscle acetylcholine receptor (AChR-MG), whereas about 5% of MG patients have autoantibodies against the muscle specific kinase (MuSK-MG). In the remaining about 10% of patients no autoantibodies can be found with the classical diagnostics for AChR and MuSK antibodies (seronegative MG, SN-MG). Since serological tests are relatively easy and non-invasive for disease diagnosis, the improvement of methods for the detection of known autoantibodies or the discovery of novel autoantibody specificities to diminish SN-MG and to facilitate differential diagnosis of similar diseases, is crucial. Radioimmunoprecipitation assays (RIPA) are the staple for MG antibody detection, but over the past years, using cell-based assays (CBAs) or improved highly sensitive RIPAs, it has been possible to detect autoantibodies in previously SN-MG patients. This led to the identification of more patients with antibodies to the classical antigens AChR and MuSK and to the third MG autoantigen, the low-density lipoprotein receptor-related protein 4 (LRP4), while antibodies against other extracellular or intracellular targets, such as agrin, K_v_1.4 potassium channels, collagen Q, titin, the ryanodine receptor and cortactin have been found in some MG patients. Since the autoantigen targeted determines in part the clinical manifestations, prognosis and response to treatment, serological tests are not only indispensable for initial diagnosis, but also for monitoring treatment efficacy. Importantly, knowing the autoantibody profile of MG patients could allow for more efficient personalized therapeutic approaches. Significant progress has been made over the past years toward the development of antigen-specific therapies, targeting only the specific immune cells or autoantibodies involved in the autoimmune response. In this review, we will present the progress made toward the development of novel sensitive autoantibody detection assays, the identification of new MG autoantigens, and the implications for improved antigen-specific therapeutics. These advancements increase our understanding of MG pathology and improve patient quality of life by providing faster, more accurate diagnosis and better disease management.

## Introduction

Myasthenia gravis (MG) is an antibody-mediated autoimmune disorder affecting skeletal muscles, characterized by fluctuating muscle weakness and abnormal fatigability. MG is caused by autoantibodies, which target proteins of the neuromuscular junction (NMJ), damaging the postsynaptic muscle membrane and impairing signal transmission from motor neurons to the muscle (1, 2).

**Figure 1:**
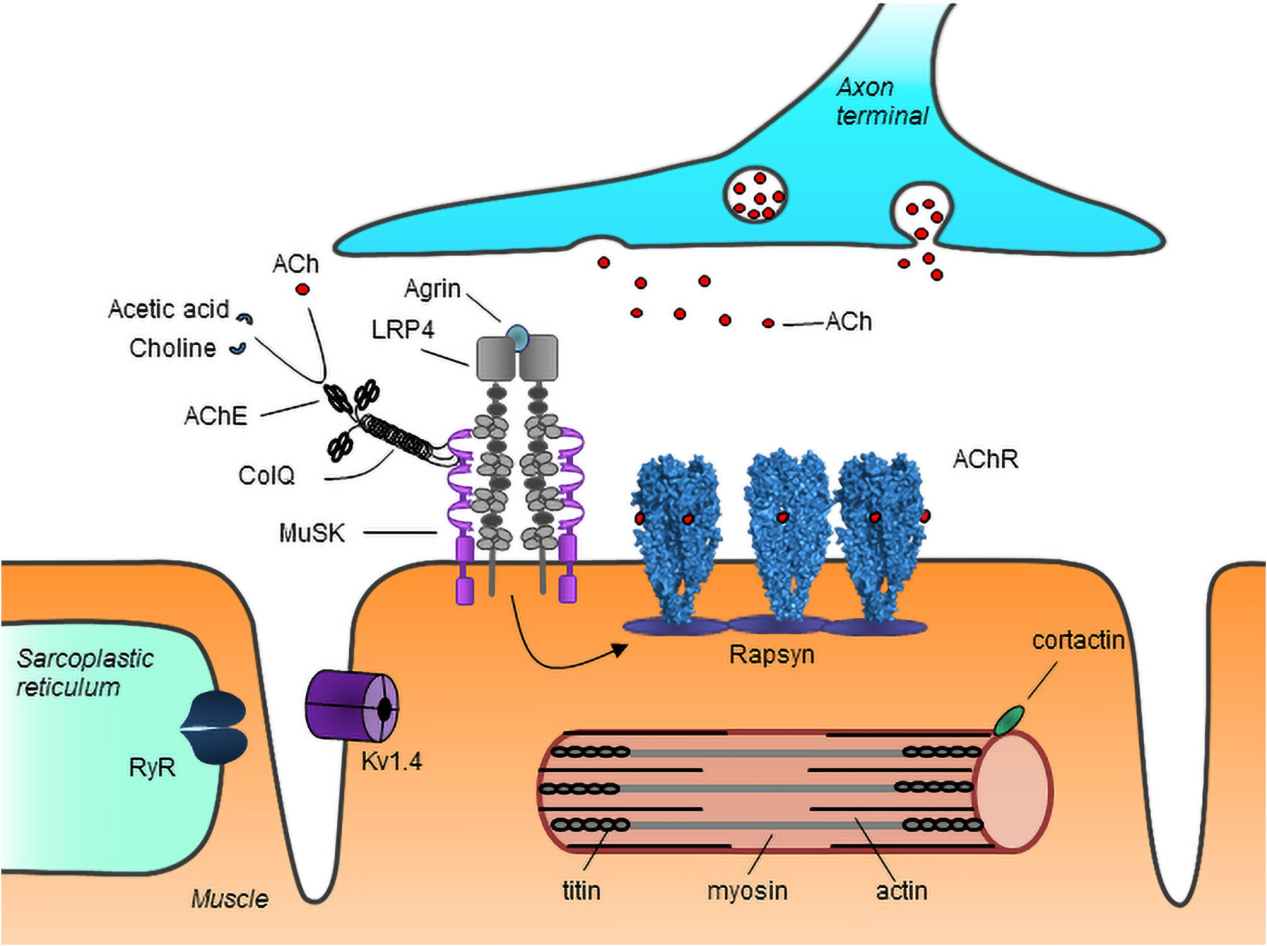
Schematic representation of major neuromuscular junction and myotube proteins targeted by autoantibodies in MG. Neuron-released agrin activates LRP4 on the muscle membrane, initiating a pathway which via MuSK leads to rapsyn-dependent AChR clustering at the NMJ. Acetylcholine (ACh) released from the nerve terminal binds to AChRs causing their activation. ACh is broken down by AChE into choline and acetate, thus terminating its action. AChR, acetylcholine receptor; MuSK, muscle specific kinase; LRP4, low-density lipoprotein receptor-related protein 4; RyR, ryanodine receptor; ColQ, collagen Q; AChE, acetylcholinesterase; Kv1.4, voltage gated potassium channel 1.4. Image from Lazaridis and Tzartos (5).

The organization of the NMJ is crucial for effective signal transmission (3, 4). Acetylcholine receptors (AChRs) on the muscle cell membrane bind acetylcholine released from the axon terminals and open to allow inflow of ions, which leads to depolarization of the membrane. The AChRs are clustered at the NMJ resulting in a localized high density of receptors, which ensures the efficiency of signal transmission. Neural agrin, released from nerve terminals, binds to low-density lipoprotein receptor-related protein 4 (LRP4) on the muscle membrane, activating it to form complexes with muscle specific kinase (MuSK). This results in the phosphorylation and activation of MuSK, which in turn leads to rapsyn-mediated AChR clustering at the NMJ (Figure 1).

MG is heterogeneous in terms of symptom presentation, as well as pathophysiology, since different proteins of the NMJ can be targeted (6, 7). MG symptoms usually manifest initially at the ocular muscles and in ~15% of patients they remain localized, commonly referred to as ocular MG (OMG). In the majority of patients, however, the symptoms progress within a couple of years to other skeletal muscles leading to generalized MG (GMG). In terms of age of onset the disease presents with two peaks of incidence: the first well below the age of 50, termed early-onset MG (EOMG), more commonly affecting women and the second above the age of 50 (late-onset MG, LOMG) more common among men.

Although MG is a relatively rare disease with a prevalence of 150–300 per million population and an incidence of ~10 per million per year (8), it is considered a model antibody-mediated autoimmune disorder, due to the extensive characterization of the main autoantibodies and target antigens. In more detail, in most patients (~85%) the autoantibodies target the muscle AChR. In ~6% of patients the autoantibodies target MuSK, while autoantibodies targeting LRP4 are found in about 2% of MG patients. The pathogenicity of autoantibodies has been demonstrated by the improvement of patients' symptoms following plasmapheresis and by the onset of passive transfer experimental autoimmune MG (EAMG) when they are injected into experimental animals (9–13). Patients without detectable autoantibodies are referred to as seronegative (SNMG). Some MG patients have antibodies against a number of other extracellular or intracellular targets. Although the pathogenicity of these autoantibodies is often uncertain or unlikely, they can still be very valuable as disease biomarkers.

The detection of autoantibodies is crucial for MG diagnosis and for the differential diagnosis of many disorders with similar presentation. We will review the main autoantibodies found in MG, their implications for diagnosis and patient stratification, and recent advances for improved diagnostics based on more sensitive tests or the discovery of new target antigens. In addition, we will present an overview of recent efforts to develop targeted therapeutic methods aiming only at the antigen-specific components of the immune system, further highlighting the importance of autoantigen determination in MG diagnosis.

## MG Classification Based on Autoantibody Specificity

### Patients With AChR Antibodies

AChRs are located at the end plate of the muscle post-synaptic membrane, opposite the axon terminals. They are composed of five homologous subunits with a stoichiometry of α_2_β*δε* in adult and α_2_β*γδ* in fetal or adult denervated muscles (14). The autoantibodies target the N-terminal extracellular domains (ECDs) of the AChR subunits. About half of the autoantibodies bind the AChR α subunit and studies in experimental rats have suggested that these are the most pathogenic (10). A region of the α subunit composed mainly by amino acids 67–76 with some contribution from other segments has been identified to be particularly targeted, commonly referred to as the main immunogenic region (MIR) (15–17). However, autoantibodies against all five subunits, including the γ subunit of the fetal AChR, can be found, even in the same patient (18–21).

AChR antibodies confer their pathogenicity by three mechanisms. Firstly, they can activate the complement cascade, since they belong mainly to the IgG1 and IgG3 subclasses, thus causing destruction of the post-synaptic membrane (22, 23). The loss of the typical local architecture results in a severely diminished efficiency of signal transduction between nerve and muscle. Secondly, they can cross-link AChRs causing their internalization and destruction by a process called antigenic modulation, thus leading to a reduction in the number of functional receptors in the post-synaptic membrane (24). Lastly, antibodies that bind close to the AChR ligand binding site are thought to directly block acetylcholine binding and receptor activation (25).

The detection of serum AChR antibodies has been an invaluable tool in the diagnosis of MG. Although the AChR antibody titer does not correlate with disease severity across different MG patients, the temporal variation of titers from individual patients appear to be associated not only with symptom severity but with response to treatment as well (26). Therefore, in addition to diagnosis, AChR antibody measurement can be useful for MG patient monitoring. Nonetheless, in very rare cases AChR antibodies can be found in non-MG patients with other autoimmune disorders or with thymoma (27).

### Patients With MuSK Antibodies

MuSK is a key player involved in NMJ organization and maintenance. It is located on the muscle membrane where it interacts with LRP4 propagating the signal for AChR clustering, while it is involved in tethering acetylcholisteresase (AChE) via interactions with collagen Q (ColQ). MuSK is a transmembrane protein with an extracellular domain containing three immunoglobulin-like regions and a frizzled-like region, a transmembrane helix domain and a cytoplasmic domain with tyrosine kinase activity. Most of the MuSK antibodies are directed against the immunoglobulin-like regions of the extracellular domain (28, 29). This binding appears to block the interactions of MuSK with LRP4 or ColQ resulting in reduction of both agrin-dependent and agrin-independent AChR clustering (30–32). Antigenic modulation and complement activation are not thought to be significant in pathology, since MuSK antibodies are mostly of the IgG4 subclass, which does not activate complement and is functionally monovalent (33, 34). Nonetheless, since IgG1-−3 MuSK antibodies are also present in patients' sera, they could have pathogenic activity, although their relative contribution remains unclear.

MuSK antibodies are found in about 6% of MG patients, accounting for 40% of patients without AChR antibodies. However, their prevalence varies among countries possibly due to genetic and environmental factors, with northern European countries presenting lower rates than those in south Europe and the Mediterranean (29, 35–39), while in Japanese populations they are even less common with a prevalence of 2–3% (40). Similarly to AChR antibodies, their detection is crucial for MG diagnosis and monitoring. Interestingly, their titer has been shown to positively correlate with symptom severity not only in individual patients but in the population as well (41, 42).

### Patients With LRP4 Antibodies

LRP4 is a transmembrane protein, containing several low-density lipoprotein domains, expressed in skeletal muscles and in motor neurons in the brain. In the muscle, it binds neural agrin released from the nerve terminals initiating the signal via MuSK for AChR clustering (43). LRP4 antibodies belong mostly to the IgG1 subclass and *in vitro* they have been shown to be capable of complement-mediated cell lysis (13, 44). However, the contribution of complement activation in their pathogenicity is still unclear and the main *in vivo* mechanism at play is thought to be inhibition of interaction with MuSK, causing disruption of normal NMJ organization (13, 44–46).

The overall prevalence of LRP4 antibodies in MG patients appears to be around 2% [i.e., ~19% of SNMG patients (47)], although there was considerable variation among initial studies with reported rates of 2–45%, possibly due to differences in the detection method used, the source of the antigen (animal or human) and the populations studied (44–46). A lower prevalence has been reported among Chinese MG patients accounting for 0.8–1.7% of total and 1–2.9% of SNMG patients (48, 49). Interestingly, LRP4 antibodies have also been reported in 10–23% of amyotrophic lateral sclerosis (ALS) patients (50, 51) and in 3.6% patients with other neurological diseases but not in healthy controls (47). Despite their frequent detection in ALS, their detection is a significant aid in MG diagnosis in parallel with the clinical presentation of the patients.

### Patients With Other Antibody Specificities

In addition to the main MG antibody specificities discussed above, a number of other autoantibody targets, both extracellular and intracellular, have emerged in MG patients.

#### Extracellular Antigens

Activation of the LRP4/MuSK complex to drive AChR clustering is caused by neural agrin. Agrin antibodies have been detected in 2–15% of MG patients, though in most cases they were also positive for antibodies against AChR, MuSK, or LRP4 (52–55). Agrin antibodies have also been found in 14% of ALS patients (50). However, they have been shown to inhibit agrin-induced MuSK activation *in vitro*, and immunization with neural agrin caused MG symptoms in experimental animals, suggesting that these antibodies are involved in MG pathology (54, 56). Their detection can be valuable for disease management, as they have been shown to be associated with moderate to severe symptoms and moderate response to treatment (52).

In some MG patients antibodies against the voltage gated potassium channel α-subunit Kv1.4 have been found, which in addition to the central nervous system is expressed in skeletal and heart muscles. A prevalence of 11–18% among MG patients has been reported, although the associated symptom severity appears to depend on the population studied. In a Caucasian patient cohort Kv1.4 antibodies were associated with LOMG patients and mild disease, often remaining purely ocular (57), while in Japanese patients they correlated with increased disease severity, myasthenic crises and the presence of thymoma (58–60). Furthermore, since in the Japanese cohort myocarditis or abnormal ECG findings were present in as many as 27 and 60%, respectively of Kv1.4 antibody positive patients, they could be an important marker of myocarditis or cardiac dysfunction among Japanese MG patients.

The activity of acetylcholine on AChRs is controlled by the enzyme AChE, which breaks down acetylcholine to choline and acetate thus terminating its action. AChE is located close to the postsynaptic membrane, where it is anchored on MuSK via molecules of ColQ (61). Antibodies against both AChE and ColQ have been found in some MG patients. AChE antibodies have been reported in 5–50% of MG patients, but they are not specific for MG since they are also found in many patients with other autoimmune diseases, while no correlation has been identified with clinical characteristics or symptoms (62–64). ColQ antibodies have so far been detected in about 3% of MG patients, including among SNMG, although again they do not appear to be MG specific and no evidence of pathogenicity has been found yet (65). Finally, antibodies against collagen XIII, a transmembrane collagen, have been detected in the serum of about 7% of MG patients with AChR antibodies and 16% of SNMG, but their presence did not correlate with symptom severity (66). Furthermore, they too are not specific for MG, since they are also found in patients with Grave's ophthalmopathy (67). Overall, the lack of MG-specificity of AChE, ColQ, and collagen XIII antibodies as well as the lack of association with clinical characteristics, which might have attributed a prognostic value, make the usefulness of these antibodies in MG diagnosis uncertain and further investigation is required.

#### Intracellular Antigens

The first autoantibodies, after AChR antibodies, to be identified in MG were the striational antibodies, named after the characteristic staining patterns produced in sarcomere sections by patients' sera. The term in fact collectively refers to several antibodies directed against different muscle proteins including titin, the ryanodine receptor (RyR), actin, myosin, tropomyosin, filamin, and others (68–71). Although the pathogenicity of these antibodies is unlikely, due to the intracellular localization of their target antigens, the diagnostic and prognostic value for titin and RyR antibodies has long been established.

Titin is the largest protein known to date, a filamentous molecule with a molecular weight of up to 4,200 kDa (72). Interestingly, titin antibodies only bind to a 30 kDa domain, called MGT30, located near the A/I band junction (73). Until recently, titin antibodies were only found in MG patients with AChR antibodies, being detected in 20–40% of them. These antibodies show a strong correlation with disease age of onset, since they are present in about 6% of EOMG but 50–80% of non-thymomatous LOMG patients (74–78), but in 50–95% of EOMG with thymoma and only few non-thymoma patients, so their presence provides a strong indication for thymoma (69, 75, 78–82). Additionally, they appear to be prognostic of more severe disease in all age groups (78, 80, 81, 83). More recently, low-titer titin antibodies were detected in SNMG as well (84). These low titin antibody titers did not correlate with the presence of thymoma, in accordance with previous findings that thymoma is unlikely in MG patients without AChR antibodies (82).

RyR is a transmembrane protein forming a calcium channel in the sarcoplasmic reticulum, where it mediates Ca^2+^ release into the cytoplasm, facilitating muscle contraction in response to stimulation. Similarly to titin antibodies, RyR antibodies are found in few EOMG but in up to 40% of LOMG patients, while they are found in 75% of thymomatous MG patients and their presence is prognostic of more severe disease progression (81, 85–88).

Rapsyn is a scaffolding protein, which in the muscle plays a role in AChR clustering by linking the intracellular domains of the receptors (89). Antibodies against rapsyn have been found in about 15% of MG patients, including among SNMG (90). However, rapsyn antibodies have also been found in various other autoimmune disorders decreasing their value as MG specific diagnostic markers, while no correlation with disease severity has been identified (91).

Cortactin is a cytoplasmic protein also involved in AChR clustering downstream of MuSK. Cortactin antibodies have been detected in about 9.5% of AChR antibody positive MG patients and 24% of SNMG patients, while they seem to be associated with mild disease (92–94). Nonetheless, their importance for MG diagnosis is still unclear, since they are also found in ~12.5% of patients with other autoimmune diseases and 5% of healthy controls (93), as well as up to 26% of patients with polymyositis, dermatomyositis, and immune-mediated necrotizing myopathy (95).

#### Relevance in MG Diagnosis

Although involvement in pathogenicity of most of the above antibodies against extracellular targets is often not clear yet, their detection can be valuable for MG diagnosis, especially in the case of otherwise seronegative patients. However, further validation or improvement of the detection assays is necessary, since in many cases they appear to lack good specificity for MG. The detection of antibodies against intracellular antigens, has proven invaluable as markers of disease severity, or identification of comorbidities, such as titin antibody detection for thymoma in EOMG. Furthermore, these antibodies, although unlikely to be pathogenic themselves, can play a significant role in diagnosis of SNMG patients, where the pathogenic autoantibodies may not be detectable by current assays, like anti-titin antibodies detected by RIPA in AChR-seronegative patients (84).

## Methods for Serological Diagnosis of MG

Serological tests for the detection of autoantibodies play a vital role in MG diagnosis. Being minimally invasive methods, they do not present a major barrier for testing and a single serum sample could potentially be tested by several assays if required, without the need for repeated hospital visits by patients. Although a final diagnosis may rely on additional tests, such as electrophysiological examination or assessment of response to AChE inhibitors, the high specificity of many MG antibody assays considerably facilitates diagnosis (Table 1).

**Table 1:** Autoantibody specificities in MG with clinical associations and common detection assays used.

	**Target antigen**	Detection assay^*^	**Clinical presentation**	**References**
Extracellular	AChR	RIPA: Good specificity (~99%) and sensitivity (~85% for GMG and ~50% for OMG). Requirement for specialized equipment and use of radioactivity.	The major MG subgroup. Practically all MG symptoms may be present. The presence of AChR antibodies is very rare in other diseases. Thymic abnormalities (mostly thymic hyperplasia) are common, and thymoma in ~10% of patients.	Several references, including (7, 96–99)
		ELISA: Various assays developed with reported specificities ranging between 96.1 and 99% and sensitivity for GMG 79.5–91.5%. Easier to adopt in non-specialized laboratories.		
		CBA (clustered AChR): Allows detection of antibodies bound only to high density AChRs, or those whose epitopes are altered during receptor solubilization. Detection of ~20% of previously SNMG. Requirement for specialized equipment.		
	MuSK	RIPA: very good specificity. Detection of antibodies in ~40% of AChR antibody negative patient	Usually manifested by bulbar symptoms. Moderate to severe symptoms. No thymic abnormalities.	(29, 37, 40, 100, 101)
		ELISA		
		CBA: Detection of 8–13% of patients negative for AChR and MuSK antibodies by RIPA. Can detect up to ~99% of RIPA-positive samples and has ~100% specificity when IgG Fc-specific 2nd antibodies are used.		
	LRP4	ELISA	Milder symptoms than AChR antibody positive MG. No thymoma.	(47, 50)
		CBA: Detection in ~6–19% of SNMG patients, but also in 10–23% of ALS patients.		
	Agrin	ELISA or CBA: Detected in up to 15% of MG patients, mostly seropositive. They have also been found in 14% of ALS patients.	Associated with more severe symptoms and moderate response to treatment.	(50, 53)
	Kv1.4	Immunoprecipitation of ^35^S-labeled cells extracts followed by SDS-PAGE.	In Japanese patients they are associated with more severe disease and myocarditis, while in Caucasian patients they are associated with LOMG and mild symptoms	(57, 59)
	AChE	ELISA: 5–50% of MG patients positive, but also several patients with other autoimmune diseases.	No association with thymic pathology and symptom severity.	(63, 64)
	ColQ	CBA: Found in ~3% of MG patients, but lack specificity.	Not determined.	(65)
	Collagen XIII	ELISA: Found in ~16% of SNMG. They are also associated with Grave's ophthalmopathy.	No association with disease severity apparent.	(66, 67)
Intracellular	Titin	ELISA: Detection of titin antibodies only in AChR Ab positive MG.	More common in LOMG, rare in non-thymomatous EOMG, but present in 50–95% of EOMG with thymoma. Their presence corelates with increased symptom severity	(69, 71, 78, 81, 84)
		RIPA: Detection of titin antibodies in all MG subgroups, including 13.4% of SNMG (low titers).	MG biomarker in “seronegative” MG. Low titer antibodies detected by RIPA αre not prognostic of more severe disease or thymoma.	
	RyR	Immunoblots or ELISA: Detection of RyR antibodies only in AChR Ab positive MG.	Present in 75% of thymomatous MG patients. Their presence corelates with increased symptom severity.	(85, 88)
	Rapsyn	Immunoblots: Detected in ~17% of SNMG, but they were also detected in 10 and 78% of OND and SLE patients, respectively.	No association with disease severity apparent.	(90)
	Cortactin	ELISA or Western blot: Detected in up to 24% of SNMG, but not specific–also present in 12.5% of other autoimmune diseases and up to 26% of myositis patients.	They have been reported to be prognostic of mild disease.	(92, 93)

* *Not all assay are available for routine diagnosis yet*.

Radioimmunoprecipitation assays (RIPA) are to this day the golden standard of serological MG tests, due to their high sensitivity and their ability to provide quantitative data allowing detailed patient monitoring. RIPAs are widely applied for the detection of AChR, MuSK, and, less frequently, other antigens. The AChR antibody assay is based on indirect labeling of solubilized AChR with ^125^I-α-bungarotoxin, a highly specific AChR antagonist (102, 103). AChR can be obtained from human muscle from amputees or, currently more common, from AChR-expressing cell lines, such as CN21, which have been engineered to express both the fetal and adult types of the receptor, thus also detecting antibodies against the AChR γ subunit (104). The wide use of the AChR RIPA owes to the ~99% specificity of the assay and its high sensitivity, which amounts to about 85% among GMG patients and 50% for OMG (105). In fact, many of the “seronegative” by AChR RIPA OMG patients have been found positive by other assays and/or for other antigens including: cell based assay (CBA) for AChR clusters [up to 50% (96)], for LRP4 [up to 27% (47)], for MuSK [16% (100)], or RIPA for titin antibodies [12% (84)] with some double positives; yet a few false-positives have been also referred by these assays. It is unknown whether the remaining “seronegative” OMG patients are true seronegative or have yet undetectable antibodies to known or yet unknown antigens. The fact that those OMG patients with AChR antibodies have generally low antibody titers may suggest that some of the remaining “seronegative” have yet undetectable AChR antibodies. Assays for the detection of blocking antibodies, i.e., antibodies that bind to the receptor binding site, which may not be detected by the conventional RIPA, have been developed and are also commercially available. The added value from the use of these assays is limited since most patients will have non-blocking antibodies as well, while ACh binding competition appears to be less important for pathogenesis compared to complement activation. MuSK antibodies are commonly detected using directly ^125^I-labeled MuSK, with very high specificity for MG (106). The detection of AChR and MuSK antibodies in the same patient by RIPA is rare (107, 108). Recently, we developed a RIPA for the detection of titin antibodies with ^125^I-labeled MGT30 and used it to test a large cohort of samples from European MG patients, including 372 SNMG, which do not usually have detectable titin antibodies by current methods. We found that 13.4% of SNMG patients had titin antibodies, as well as 14.6 and 16.4% of patients with MuSK and LRP4 antibodies, respectively (84). The RIPA-detected titin antibodies in SNMG were not predictive of more severe disease. Nonetheless, titin antibodies detected by RIPA are a valuable biomarker for the diagnosis of otherwise SNMG patients.

Efforts to improve the sensitivity of the classical RIPA have resulted in the development of modified assays, using much larger serum volumes in order to detect antibodies at lower titers. Different approaches have been explored in order to minimize non-specific binding, which would render the use of large serum volumes impossible. In the case of AChR antibodies, semi-purified anti-human IgG was used as secondary antibody, allowing an increase of serum volumes by 16-fold and consequently reducing the positivity titer cut-off value from 0.5 to 0.1 nM (109). The application of this method allowed the detection of AChR antibodies in 20 of 81 previously SNMG patients tested. For MuSK antibody detection a two-step approach has been proposed, initially semi-purifying the MuSK antibodies by affinity chromatography with sepharose-immobilized MuSK and then using the concentrated antibodies for standard RIPA (110). This modification allowed the use of up to 50 times larger serum volumes for the assay, which resulted in the detection of previously SNMG patients, without a compromise in specificity.

Enzyme-linked immunosorbent assays (ELISAs) have also been in use for the detection of AChR and MuSK antibodies, though less commonly than RIPA (97, 111). The ELISA has advantages as it does not involve the use of radioactivity and can be performed with standard equipment in most laboratories. For AChR antibodies, different ELISAs have been developed, either directly coating AChR onto ELISA plates followed by serum incubation, or by preincubation of AChR with serum in solution followed by measuring the inhibition of binding to a set of AChR monoclonal antibodies (immobilized and in solution). Although some studies have found the ELISA as specific and at least as sensitive as the RIPA, in others the ELISA presents with lower specificity and sensitivity, perhaps explaining its limited adoption (97, 98, 111). Assays aiming at the detection of modulating or blocking antibodies have also been developed, but they did not improve the sensitivity significantly compared to the standard RIPA (112, 113). On the other hand, ELISA with immobilized titin MGT30 domain is currently the most widely used method for the detection of titin antibodies. Other antibodies usually tested for by ELISA include cortactin and RyR antibodies using as antigen recombinant protein domains (114).

Several efforts have been made to produce other non-radioactive alternatives to RIPA with comparable sensitivity. A promising solution appears to be fluorescence immunoprecipitation assay (FIPA), which involves labeling of the target antigen with a fluorescent dye. In one approach for AChR labeling, the α, γ, and ε subunits were tagged with EGFP, before transfection together with the remaining subunits into HEK293 cells, while for MuSK the extracellular domain only was used labeled with maccherry and expressed in insect S2 cells (115). The overall sensitivity was shown to be very close to that of the RIPA for both AChR and MuSK antibodies. Furthermore, by labeling each antigen with a different fluorescent dye both AChR and MuSK antibodies could be detected simultaneously in the same assay, thus potentially reducing the cost and time for diagnosis. A similar method based on labeling recombinant fragments of the AChR α subunit with *Renilla luciferase* has been developed with good specificity (97%), but it was able to detect AChR antibodies only in 32% of MG patients, potentially due to the use of part of the α subunit rather than whole AChR (116). Further investigation with respect to the diagnostic value of assays employing AChR fragments is necessary.

The application of CBAs in MG diagnosis has been expanding over the last years. The method involves the transient or stable expression of the target antigen in a cell line, followed by incubation of the cells with test serum and the detection of autoantibody binding by fluorescence microscopy using labeled secondary or tertiary antibodies.

In the case of AChR antibody CBA, co-transfection of the cells with rapsyn, in addition to the AChR subunits, induced clustering of the receptors, thus permitting detection of antibodies that bind only to high density AChRs mimicking their clustering at the NMJ, or of antibodies whose epitopes are altered by the detergent solubilisation of membranes during the isolation of AChR antigen. Despite initial reports of high seropositivity found among SNMG with CBA for AChR antibodies (96, 99, 100, 109, 117, 118), routine diagnosis suggests that the overall frequency of antibodies against clustered AChRs in SNMG patients is around 20% or less (100, 119). Autoantibody titration can be achieved by using serial dilutions of sera, but based on our experience with both assays, it cannot reach the accuracy of the RIPA. Nonetheless, CBA has become invaluable for the diagnosis of SNMG patients, with several studies reporting detection of AChR antibodies that were undetected by other current diagnostics (115, 118, 120, 121). On the other hand, even for sera found positive for AChR antibodies by RIPA, a CBA test could be useful to confirm that the detected antibodies bind on the cell embedded AChR. The use of both fetal and adult forms of the AChR not only appears to increase the sensitivity of the assay, but also enables the discrimination among fetal or adult AChR directed antibodies (122). The latter is important for the diagnosis of transient neonatal MG not associated with maternal MG, a condition arising from the presence in the mother of antibodies against only the fetal AChR, which may not cause MG symptoms in the mother but can be detrimental for the new-born (20, 123, 124).

CBAs have also contributed significantly in the detection of MuSK and LRP4 antibodies in previously SNMG patients, including Asian populations where MuSK-MG is less common (99, 115, 120, 125). We have used CBAs for MuSK and LRP4 to test a cohort of sera from 13 European countries including over 630 samples from SNMG patients. We found that about 13% of SNMG samples were positive for MuSK antibodies, with a variation in the rates among countries, ranging from 5–22% (100). The MuSK CBA has allowed the detection of antibodies in SN-OMG patients as well, which is not common with RIPA (100, 115). Of note, most of the MuSK antibodies detected belonged to the IgM rather than IgG class. Using the LRP4 CBA, 19% of SNMG were found positive for LRP4 antibodies with an inter-country variability of 7–33% (47). The percentage of patients positive for more than one antibody specificities has increased by the use of CBAs. In more detail, 7.5% of AChR antibody positive and 15–20% of MuSK antibody positive sera have also been found positive for LRP4 antibodies, while 0.5–12.5% AChR antibody positive patients were reported positive for MuSK antibodies as well (47, 48, 52, 100).

Although the presence of antibodies only detectable by CBA is associated with milder disease and better response to treatment (118), these antibodies have also been shown to be pathogenic. Indeed, antibodies against clustered AChRs belong to the complement-activating subclasses and cause complement depositions on the cell surface (96). Furthermore, MuSK IgG antibodies, but not IgM, detected by CBA were shown to inhibit agrin-induced AChR clustering on the surface of C2C12 myotubes (101).

The specificity of the secondary antibodies and by extension the antibody classes detected by CBA appears to be important (119). For example, anti-human antibodies directed against the intact light and heavy IgG chains can also bind to IgM, and possibly other antibody classes as well. A study using such secondary antibodies for MuSK CBA resulted in a significant decrease in specificity (11 and 19% positives among the healthy and disease controls, respectively), as well as in sensitivity (101). On the other hand, the use of a secondary antibody specific for the Fc part of the IgG heavy chain, which does not cross-react with other Ig classes, resulted in the detection of 99% of MuSK RIPA positive samples and 100% specificity, although this was accompanied by a decrease in the number of positives among SNMG (101). Since IgM may not be pathogenic, the importance of discrimination of the antibody classes for diagnosis remains to be fully assessed.

Recently, a modified CBA approach was developed based on the generation of stably-transfected HEK293 cell with the target antigen and, following incubation with the test sera, autoantibodies were measured by FACS analysis, providing more quantitative results. The assay has been used for the detection of antibodies against various antigens such as Kv1.4 and even the intracellular titin (126). In fact, the cytometric CBA showed improved sensitivity compared to the ELISA for titin. Furthermore, it could facilitate the diagnosis of Kv1.4 antibodies despite the somewhat lower sensitivity compared to the currently used method, which is relatively complicated and laborious, involving the immunoprecipitation of ^35^S-labeled cell extracts from rabdomyosarcoma and leukemic cells followed by electrophoresis analysis. The presence of a 70 kDa Kv1.4 protein band in the former but not the latter extracts is considered a positive finding (59).

A significant disadvantage of most of the aforementioned methods is the requirement of specialized equipment and expertise. Efforts are made for the development of fast, easy to perform and instrument-free assays. The use of such assays in decentralized small clinics and doctors' offices could reduce the time to diagnosis significantly, improving disease management. To this end, we have developed an assay based on the immobilization of antigen on a stick-type solid surface (immunostick) at high density. The immunostick can be immersed in succession into the undiluted test serum, secondary antibodies and substrate solution, similar to standard ELISA, but with much reduced incubation times, allowing completion in less than an hour. Furthermore, immobilization of various antigens in different zones of the immunostick could allow the simultaneous detection of more than one MG autoantibodies. Evaluation of this method for the detection of AChR antibodies, showed that it had very good specificity and sensitivity (99 and 91%, respectively) (127). A similar approach based on a modified dot-blot method, using AChR preparations immobilized onto nitrocellulose membrane, achieved the same sensitivity as the ELISA (128).

## Development of Therapies Based on Autoantigen Specificity

In addition to its value for MG diagnosis, the determination of autoantibody specificities is important for efficient management of the disease. For example, the differentiation among AChR and MuSK antibody positive patients has important implications for therapy, since the latter can present with adverse effects when treated with AChE inhibitors, a common first line AChR-MG treatment, thymectomy, or the use of complement inhibitors does not appear to be beneficial to them (129). On the contrary, MuSK antibody positive patients usually respond very well to rituximab or therapeutic plasma exchange (130–132). Of note, the detection of any autoantibody specificity could provide an indication for the use of neonatal Fc receptor (FcRn) inhibitors, which work by blocking IgG recycling via the FcRn, thus reducing IgG half-life and which show potential for MG treatment in recent clinical trials (133).

Current common treatments for MG include AChE inhibitors, immunosuppressive drugs, thymectomy, intravenous immunoglobulin (IVIG) and plasmapheresis (1, 134). However, these approaches are to a large extent not specific and can thus be accompanied by various side effects. The problem is augmented given the long-term immunosuppression that may be required, increasing the risk of infections or neoplasia. Furthermore, a number of patients may remain unresponsive to current treatments (132). The development of antigen-specific therapies targeting only the pathogenic components of the immune system would, therefore, greatly benefit MG patients. Knowledge of the autoantibody repertoire of each patient is vital for such approaches to be implemented, further underlining the role of serological diagnostics.

**Figure 2:**
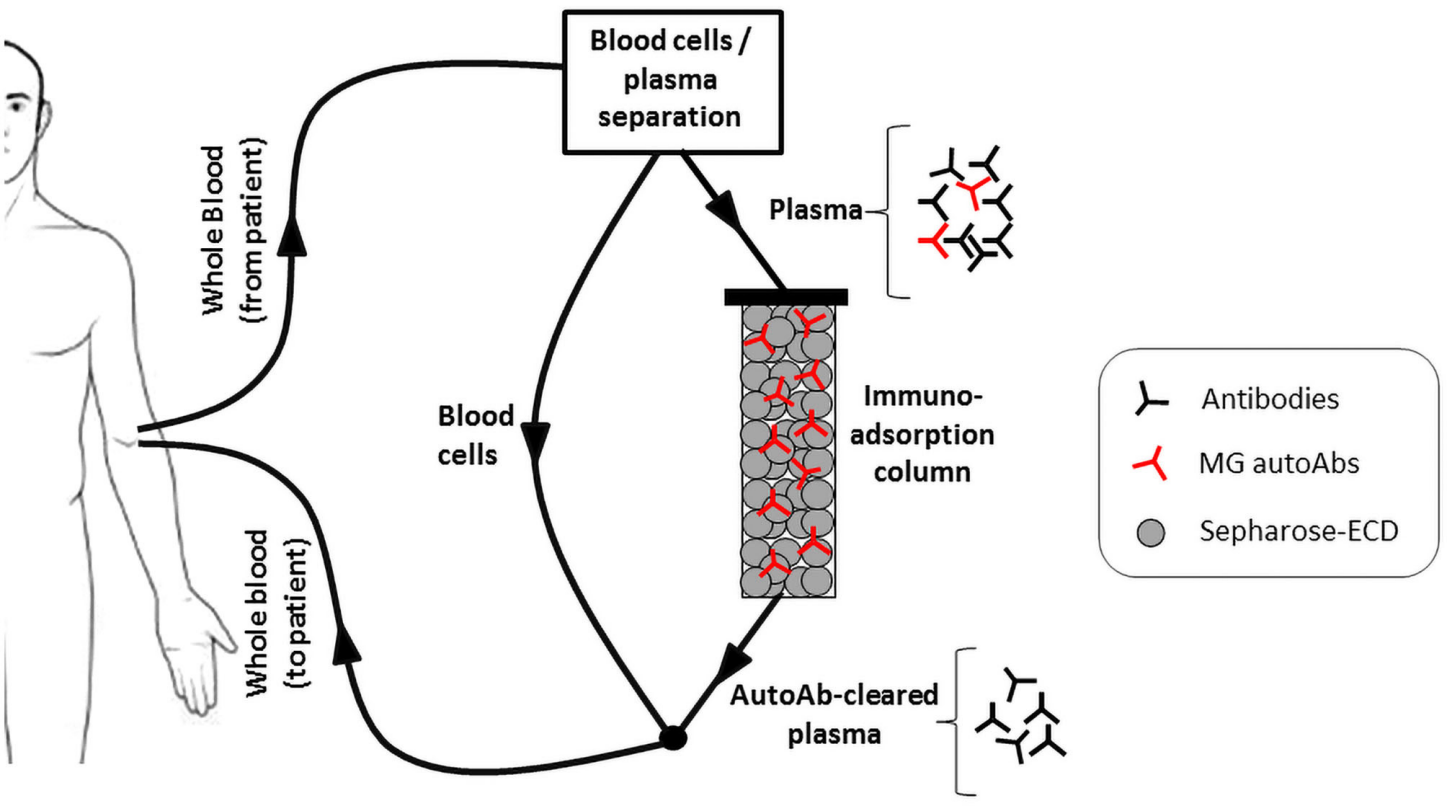
The antigen specific immunoadsorption therapy approach. Recombinant extracellular domains (ECDs) of the AChR or MuSK are immobilized onto sepharose and packed into a column. During treatment, the patient's plasma is passed through it, allowing the selective binding and removal of only the MG autoantibodies. The autoantibody-depleted plasma is then returned to the patient.

One approach would be antigen-specific immunoadsorption, which is based on the selective removal of the autoantibodies from the patient's circulation. The procedure is a modification of plasmapheresis, whereby the isolated plasma, instead of being discarded, is passed through a matrix allowing the removal of the autoantibodies, before being returned to the patient (135) (Figure 2). Since no replacement fluids would be needed as in plasmapheresis, an additional advantage of the approach will be the reduction in risk of infection or allergic reactions. Efforts to develop such a matrix have been made by immobilization of recombinant extracellular domains of AChR or MuSK onto sepharose. Expression of the recombinant proteins has been optimized to achieve sufficient production yield and purity together with maximum antibody binding (110, 136). A number of *in vitro* experiments have established the efficiency, speed and specificity of AChR or MuSK autoantibody binding from sera of immunized experimental animals or MG patients (28, 137). Especially in the case of MuSK antibodies, immunoadsorption resulted in almost complete removal of the autoantibodies from all the patient sera tested. Furthermore, *ex vivo* immunoadsorption has been performed in rats with experimental autoimmune MG (EAMG), induced by immunization with human AChR or MuSK ECDs. The procedures resulted in quick and significant reduction of symptom severity, without the emergence of any adverse effects (138, 139). Although such an approach would not be a permanent cure as the autoantibodies would inevitably re-emerge, it would be greatly beneficial as a treatment option, providing immediate relief from symptoms when required, such as during myasthenic crises or pre-operatively.

Another approach for antigen-specific therapy would be to induce immunosuppression or immune tolerance in a targeted manner. In this case treatment would not have an immediate impact, but it would aim at a long-lasting or permanent effect. Indeed, studies have shown that EAMG symptoms can be prevented or ameliorated by oral or nasal administration of AChR or MuSK domains (140–144). In most studies the extracellular domain of the AChR α subunit has been used, while the response was not affected by the use of syngeneic (rat) or xenogeneic (human) AChR sequences (145). The therapeutic efficacy appeared to depend on the conformation of the administered antigens, with denatured proteins having a more pronounced effect (146). In fact an α subunit domain lacking some of the B cell epitopes has been found more efficient for treatment (147), suggesting that destruction of conformation-dependent B cell epitopes was responsible for the increased efficacy of denatured antigens. Furthermore, the use of AChR peptides corresponding to dominant T cell epitopes orally has been shown to ameliorate disease symptoms (148). Interestingly, a beneficial effect was also observed when dominant T cell epitopes were administered in the form of subcutaneous immunization in the presence of adjuvant (149). Conjugation of antigen derived peptides to immunomodulating protein domains as a means of targeting has also been explored to improve treatment potency with promising results (150). In most cases of tolerance induction, a shift in the T cell responses from Th1 to Th2 and/or Th3 was involved in mediating the therapeutic effect, evidenced by changes in the respective cytokine levels, mostly reduction in IFN-g, IL-2 and IL-12 and increase in IL-10 and TGF-β expression, accompanied by changes in the AChR IgG subclass distribution (144, 151–153).

The identification of peptides derived from the human AChR α subunit as T cell dominant epitopes, lead to the construction of altered peptides with single amino acid substitutions (termed altered peptide ligands, APL), some of which were found to inhibit T cell proliferative responses *in vitro* (154). Furthermore, oral administration of a dual APL (two APL peptides in tandem) in mice with EAMG, resulted in improvement of clinical manifestations and reduction of autoantibody titers (155). The therapeutic effect was marked by downregulation of the IFN-g and IL-2, upregulation of IL-10 and TGF-β, and induction of immunoregulatory CD4+CD25+ T cells (156, 157).

A different strategy relied on the administration of peptides incorporating only the intracellular domains of the AChR subunits, which have been shown to be incapable of disease induction (158). Although oral or nasal administration of the intracellular polypeptides was able to prevent and, in some cases, treat ongoing EAMG, the effect was greater when treatment was given as subcutaneous vaccination (142, 159). The mechanism of action appears to involve diverting the immunological response away from the production of ECD-targeting pathogenic antibodies, toward epitopes of the intracellular domains, and possibly causing apoptosis of AChR-specific plasma cells (160).

## Conclusion

The clinical presentation of MG, its underlying pathophysiology and the response to treatment vary depending on the targeted autoantigens. Assays for the detection of MG autoantibodies are central in diagnosis, and they often serve as early diagnostics in cases of clinically suspected MG. Furthermore, since serological tests can identify the autoantibody specificities in MG patients, their role extends beyond disease diagnosis as invaluable tools for MG management. Patients with suspected MG but initially negative for autoantibodies, should be retested since usually the antibody titers increase and there is epitope spreading with disease progression. Nevertheless, some MG patients remain seronegative, making the discovery of novel antigenic targets or the development of more sensitive assays against known antigens invaluable. To this end, several new antigens recognized by autoantibodies in MG patients' sera have been identified over the last years, but the diagnostic relevance for most of them remains to be fully established.

RIPAs for AChR and MuSK antibodies have been the most widely used assays, owing to their very high sensitivity and specificity. The use of CBAs in routine diagnosis, mostly for clustered AChRs, MuSK and LRP4, is being slowly introduced during the recent years, contributing in reducing the number of SNMG patients. However, a significant disadvantage of CBAs currently is their limited capability of providing accurate titer information, which in addition to the lack of commercial kits, has resulted in their use mostly for patients negative by the standard RIPAs. Cytometric CBAs providing more quantitative results have already been proposed as a useful alternative, but their value for routine diagnosis remains to be assessed. Furthermore, simpler assays designed for quick instrument-free sample analysis are being developed, which should decrease the time to diagnosis and contribute to the improvement of patients' care when they become commercially available. Finally, not surprisingly due to the nature of serological tests, irrespective of sensitivity, there is currently no single assay detecting all seropositive patients. Therefore, the potential need to ultimately use different assays for the diagnosis of these few patients must not be overlooked by the clinicians.

The identification of the antigen targeted in individual MG patients, presents the unique opportunity to develop personalized antigen-specific therapies that would selectively target the autoimmune components of the immune system. Among the approaches studied are specific removal of the autoantibodies, induction of tolerance and diversion of the immune response from the targeted autoantigen. Several studies have shown their therapeutic potential, but further pre-clinical trials are required before they can progress to clinical application. The development of such personalized approaches would increase the treatment efficacy and reduce side effects, thus significantly improving the patients' quality of life, and should be the focus of further efforts.

## Author Contributions

KL and ST researched the bibliography for the review, made substantial contributions to the content, and reviewed and edited the manuscript. KL wrote the first draft. All authors contributed to the article and approved the submitted version.

## Conflict of Interest

ST has shares in the research and diagnostic laboratory Tzartos NeuroDiagnostics. The remaining author declares that the research was conducted in the absence of any commercial or financial relationships that could be construed as a potential conflict of interest.
